# The relationship between anesthesia, surgery and postoperative immune function in cancer patients: a review

**DOI:** 10.3389/fimmu.2024.1441020

**Published:** 2024-09-04

**Authors:** Rui Guo, Wen-wen Yang, Mao-lin Zhong, Pan-guo Rao, Xin Luo, Bao-zhen Liao, Xing-heng Lei, Jun-ming Ye

**Affiliations:** ^1^ Department of Anesthesiology, First Affiliated Hospital of Gannan Medical University, Ganzhou, Jiangxi, China; ^2^ Suzhou Medical College of Soochow University, Suzhou, China; ^3^ Gannan Medical University, Ganzhou, Jiangxi, China

**Keywords:** anesthesia, surgery, cancer, immune function, postoperative

## Abstract

This review comprehensively examines the impact of anesthesia and surgical interventions on the immune function of cancer patients postoperatively. Recent studies have shown that surgery and its accompanying anesthesia management can significantly influence immune function in cancer patients, potentially affecting their prognosis. This review synthesizes clinical studies and basic research to summarize the specific effects of anesthesia methods, drugs, postoperative analgesia, intraoperative transfusion, surgical techniques, and trauma extent on the immune function of cancer patients post-surgery. Additionally, this review discusses optimization strategies based on current research, aiming to refine anesthesia and surgical management to maximize the preservation and enhancement of postoperative immune function in cancer patients, with the potential to improve clinical outcomes.

## 1 Introduction

Surgical intervention remains the primary and most effective treatment approach for malignant tumors. Surgical resection can completely remove the tumor mass and is potentially curative for many patients with early-stage cancers. However, in many cases, a small number of cancer cells may still remain in the body after tumor excision. During surgical procedures, such as palpation and resection, tumor cells may be released if the tumor mass ruptures. Additionally, tumor cells can enter the bloodstream during tumor removal. Studies (1, 2) have found that circulating tumor cells can be detected in the blood of cancer patients after surgery using PCR technology. Therefore, residual cancer cells may persist in patients’ bodies after cancer surgery. Combined with surgical trauma and postoperative bed rest, a compromised immune system after surgery increases the risk of incisional and pulmonary infections. Theoretically, postoperative immune function is crucial in suppressing the development of residual cancer cells. Both surgery and anesthesia significantly inhibit postoperative immune function, which can be detrimental to recovery. Reducing these impacts to minimize immune suppression and maintain postoperative immune function is a key focus of current medical research.

## 2 Composition of the immune system and anti-tumor immunity

The immune system is composed of immune organs, immune cells, and immune-active substances. Immune organs include the bone marrow, thymus, spleen, lymph nodes, tonsils, and appendix. The immune cells consist of lymphocytes, monocytes, phagocytes, neutrophils, eosinophils, basophils, mast cells, and platelets. Among these, lymphocytes include B cells, T cells, and NK cells, with T cells accounting for approximately 65%-75% of the total lymphocyte count. Immune-active substances comprise components such as complement, immunoglobulins, interferons, interleukins (IL), and tumor necrosis factors (TNF).Within the immune-active substances, there are pro-inflammatory and anti-inflammatory cytokines. Common pro-inflammatory cytokines include IL-1, IL-6, IL-8, and TNF-α, whereas common anti-inflammatory cytokines include IL-4 and IL-10. T lymphocytes do not produce antibodies but instead exert direct effects, hence their immune function is often referred to as cell-mediated immunity. B lymphocytes function by producing antibodies that exist within bodily fluids, thus their immune response is termed humoral immunity.

The host’s anti-tumor immune response is predominantly mediated by cellular immunity, particularly through T lymphocyte subsets and natural killer (NK) cells, which play a pivotal role. These immune cells are capable of directly recognizing and attacking tumor cells by mechanisms such as the release of cytotoxins and the induction of apoptosis, thereby killing the tumor cells. In addition, immunomodulatory substances such as cytokines (e.g., interferons, interleukins) and antibodies also participate in regulating and enhancing the host’s anti-tumor immune response. Upon recognition of tumor cell surface antigens by immune cells, the immune system initiates an immune response, which includes the attack and cytotoxic action of immune cells against tumor cells. In clinical practice, the assessment of the levels and functional states of immune cells, such as CD3^+^ (representing mature T lymphocytes), CD4^+^ (which can enhance immune function), and CD8^+^ (inhibitory T lymphocytes), and the CD4^+^/CD8^+^ ratio, serves as an indirect reflection of the host’s immunological status and provides critical data for cancer treatment and prognostic evaluation.

## 3 Surgery and postoperative immune function in cancer

The intense trauma and stress resulting from surgery can significantly suppress the postoperative immune function of cancer patients. This suppression primarily impacts cellular immune function, as evidenced by reduced lymphocyte counts and cytotoxicity, diminished NK cell activity, and decreased secretion of anti-inflammatory cytokines. In contrast, humoral immune function is less affected. Study (3) has shown that local tissue damage during surgical procedures can activate the Toll-like receptor 4/nuclear factor-κB (TLR4/NF-κB) signaling pathway, triggering the release of a cascade of inflammatory cytokines. Activation of the TLR4/NF-κB pathway is associated with tumor progression, and its inhibition can exert anti-tumor effects, whereas activation promotes metastasis or recurrence of tumor cells. Greater surgical trauma leads to more pronounced suppression of postoperative immune function. Simpler or minimally invasive surgical techniques result in less suppression of postoperative immune function. Additionally, longer surgery duration tends to increase trauma and consequently exacerbate the suppression of postoperative immune function. Shi et al. (4) conducted a prospective study on patients with colorectal cancer, evaluating changes in postoperative immune function by measuring CD3, CD4, and CD8 T lymphocytes, and the CD4/CD8 ratio. They found that, compared to open surgery, laparoscopic colorectal surgery could effectively enhance postoperative immune function and reduce immunosuppression. Cho et al. (5) found that in liver cancer surgery, laparoscopic radical hepatectomy offers significant advantages over open surgery in treating hepatocellular carcinoma. This minimally invasive approach significantly reduces the impact on liver and immune function, decreases tissue damage, and improves postoperative inflammatory responses, thereby promoting faster patient recovery. A recent study (6) compared minimally invasive surgery to traditional open surgery in breast cancer patients and found that the minimally invasive approach significantly reduces immunosuppression within the first 7 days after surgery.

Enhanced Recovery After Surgery (ERAS) is a surgical treatment concept and comprehensive management model designed to minimize postoperative complications and accelerate patient recovery. ERAS encompasses preoperative, intraoperative, and postoperative phases, integrating surgical treatment, anesthesia management, postoperative analgesia, and nursing care. These interventions aim to reduce surgical stress, pain, and postoperative complications, thereby shortening hospital stays and expediting recovery. Numerous studies (7–9) have shown that implementing ERAS principles in cancer patients significantly enhances recovery and improves postoperative immune function.

## 4 Anesthetic techniques and postoperative immune function

Previous research (10, 11) has indicated that general anesthesia exerts the most substantial suppressive effect on postoperative immune function, followed by spinal anesthesia and nerve blocks, with local anesthesia showing the weakest suppression. Among the modalities of general anesthesia, a combination of general anesthesia with epidural or nerve blocks is associated with less immunosuppression compared to general anesthesia alone. Intravenous general anesthesia is found to be less immunosuppressive than inhalational general anesthesia alone (12).

Current literature suggests that combining general anesthesia with neuraxial anesthesia or nerve blocks results in less suppression of postoperative immune function than using general anesthesia alone in cancer patients. Thus, combining general anesthesia with spinal anesthesia or nerve blocks may be advantageous for maintaining postoperative immune function in cancer surgery patients. A meta-analysis (13) of nine studies showed that, compared to general anesthesia (GA) alone, combined epidural-general anesthesia (EGA) can improve the overall survival of colorectal cancer patients, particularly those with colon cancer. A large-scale retrospective study (14) demonstrated that a combination of epidural anesthesia with general anesthesia improved 3-year and 5-year survival rates in patients with ovarian cancer post-surgery. These positive effects of combined anesthesia on survival rates have also been reported in studies on other types of cancer (15–17). A clinical study (18) found that intravenous anesthesia with propofol combined with paravertebral nerve block can reduce the rate of locoregional recurrence in patients with invasive ductal carcinoma of the breast. A study (19) utilizing animal models and clinical samples, including both retrospective and prospective studies, also yielded similar results in breast cancer. Current evidence suggests that the mechanisms by which general anesthesia combined with spinal anesthesia or nerve blocks improve postoperative immune function in cancer patients are primarily twofold. First, combined anesthesia more effectively suppresses the stress response compared to general anesthesia alone. Second, it allows for a reduction in the dosage of anesthetic agents, particularly opioids, thereby minimizing the immunosuppressive effects.

In a prospective study by Cho et al. (20), propofol-based intravenous anesthesia was found to provide better protection of immune function in breast cancer patients post-surgery compared to sevoflurane inhalational anesthesia. Yu et al. (21) compared propofol and sevoflurane anesthesia in colorectal cancer surgery, finding that the propofol group had CD45RA^+^/CD45RO^+^ levels that were similar to preoperative levels immediately and 72 hours post-surgery, whereas the sevoflurane group showed significantly higher levels than preoperative levels, suggesting that propofol intravenous general anesthesia facilitates better recovery of postoperative immune function compared to sevoflurane. A large retrospective analysis (22) involving 10,696 patients showed that compared to inhalational general anesthesia, intravenous general anesthesia was associated with a significantly higher incidence of postoperative pulmonary complications and other postoperative complications varied, suggesting that intravenous general anesthesia might be the preferred modality for general anesthesia in cancer surgeries.

## 5 Anesthetic drugs

### 5.1 Opioid drugs

Opioid drugs primarily act on u-opioid receptors. Studies (23, 24) have shown that while exerting analgesic effects, opioids also inhibit the immune function of the body, suppress NK cells function, and possess potential immunosuppressive and tumor-promoting effects. These effects are particularly evident with potent opioids such as morphine, fentanyl, sufentanil, remifentanil, oxycodone, pethidine, tramadol, and hydromorphone. Morphine suppresses the immune function of gastric cancer patients by regulating the percentage of CD4^+^, CD25^+^, FoxP3^+^ regulatory T lymphocytes (Tregs) *in vitro* and increasing the CD4^+^/CD8^+^ ratio and Tregs (25). Morphine can decrease the number of Th1 cells and the Th1/Th2 ratio in patients with colorectal cancer, which may accelerate tumor invasion, recurrence, and metastasis (26). An animal study (27) indicates that sufentanil postoperative analgesia can reduce the increase of T helper 17 (Th17) cells and Tregs in a rat hepatocellular carcinoma surgical model. Furthermore, compared to morphine, sufentanil has a lesser impact on the CD4/CD8 ratio and Treg frequencies, making it more suitable for postoperative analgesia. A retrospective clinical study (28) revealed that in patients with advanced lung cancer treated with immune checkpoint inhibitors, the use of opioids may diminish the therapeutic efficacy of these inhibitors, resulting in significantly poorer clinical outcomes. Recent studies (29, 30) have also reached similar conclusions. Additionally, the indirect impact of opioids on the immune function of cancer patients involves the activation of the hypothalamic-pituitary-adrenal (HPA) axis, which inhibits the release of catecholamines while increasing the secretion of pro-inflammatory and immunosuppressive substances, such as cortisol (31, 32).

### 5.2 Intravenous anesthetics

Propofol has antitumor and immune-protective effects, and its antitumor effect may be related to its voltage-gated channels. In a large-scale retrospective study (33) involving 2856 cases, it was found that intravenous propofol anesthesia is beneficial for improving the long-term survival rate of patients undergoing gastric cancer resection. Wang et al. (34) found that propofol in pancreatic cancer patients undergoing surgical anesthesia can enhance patients’ immune function and reduce inflammation levels. A study (35) evaluated the effects of five anesthetics, including sodium thiopental, midazolam, etomidate, ketamine, and propofol, on the expression of inflammatory genes in three cell lines, colon cancer cells (Caco-2), renal proximal tubular epithelial cells (HK-2), and hepatocellular carcinoma cells (HepG2). The study found that sodium thiopental, ketamine, and propofol significantly inhibited the expression of NF-κB and its downstream cytokines IL-1β and IL-18, while also reducing TNF-α-induced inflammatory activity, whereas midazolam and etomidate did not demonstrate this anti-inflammatory effect. Midazolam (36) inhibits the upregulation of CD80 in THP-1 cells and PMDMs cells induced by lipopolysaccharide (LPS) as well as the release of IL-6 and NO, suppressing the activation of NF-κB/AP-1 and MAPKs in human THP-1 cells and inhibiting immune function (THP-1 cells are human monocytic leukemia cells, and PMDMs cells are peripheral blood monocyte-derived macrophages). Two clinical studies (37, 38) indicate that etomidate has minimal impact on postoperative immune function in patients with lung adenocarcinoma or gastric cancer. Ketamine is commonly used clinically for pediatric basic anesthesia or intravenous anesthesia. Sun et al. (39) used young rats as experimental subjects to measure immune indicators such as IL-2, IL-4, and IL-10. They found that serum IL-4 and IL-10 levels were significantly increased in the experimental group, indicating that ketamine inhibits the immune function of young rats. The combination of ketamine and morphine may decrease the CD4 percentage, CD4/CD8 ratio, and the levels of IFN-γ, IL-2, and IL-17 through the JAK3/STAT5 pathway in cervical cancer (40).

### 5.3 Inhalation anesthetics

Inhalation anesthetics have a significant impact on postoperative immune function in cancer patients. Inhalation anesthetics such as isoflurane, sevoflurane, and halothane can reduce the cytotoxicity of NK cells, thereby suppressing immune function (41, 42). Research (43) indicates that inhalation anesthetics such as sevoflurane, isoflurane, and desflurane can upregulate the expression of various proteins associated with tumor growth, migration, and metastasis in ovarian cancer cells, such as VEGF-A, MMP11, TGF-β1 and CXCR2, with desflurane having the most significant effect. These findings suggest that inhalation anesthetics may affect cancer progression by influencing the tumor microenvironment and promoting the invasive behavior of tumor cells.

However, it should be noted that the impact of inhalation anesthetics on immune function may vary depending on the type of cancer and patient condition. For example, one study (44) showed that the inhalation anesthetic sevoflurane has no significant effect on NK cell count, cytotoxic T lymphocyte (CTL) count, or apoptosis rate in breast cancer patients postoperatively. Additionally, sevoflurane does not significantly affect the co-culture of breast cancer cells, further suggesting its limited impact on postoperative immune function in breast cancer patients. Moreover, a study (45) has indicated that sevoflurane may improve immune function by inhibiting the activation of the TLR4 signaling pathway, thereby reducing the production of inflammatory mediators such as 5-lipoxygenase products and IL-10, and decreasing the expression of CD11b on neutrophils and intercellular adhesion molecule-1.

### 5.4 Dexmedetomidine

Dexmedetomidine is an auxiliary drug widely used in clinical anesthesia. It is a highly selective and efficient α2 adrenergic receptor agonist, possessing sedative, analgesic, anxiolytic, and anti-stress effects. Numerous studies (46–48) in recent years has confirmed that dexmedetomidine can effectively reduce postoperative stress-induced inflammatory responses, alleviate postoperative immune function suppression in cancer patients, and improve overall immune function. The mechanisms of action may primarily involve two aspects: reducing the dosage of other anesthesia drugs (such as opioid analgesics, inhalation anesthetics, etc.) to alleviate immune suppression, and providing analgesia, anti-stress, and anti-inflammatory responses to alleviate immune suppression in tumor surgery patients.

A meta-analysis study (47) evaluated the immunomodulatory effects of dexmedetomidine in patients undergoing digestive tract cancer surgery, finding that it could decrease postoperative levels of C-reactive protein (CRP), TNF-α, and IL-6, while simultaneously increasing the level of IL-10, indicating that dexmedetomidine contributes to mitigating postoperative systemic inflammatory responses. Furthermore, it was observed to increase the number of CD4^+^ T cells and the CD4^+^/CD8^+^ ratio, thereby improving immune function. Other research (48) found that it could elevate interferon-gamma levels, reduce the severity of early postoperative pain, and decrease opioid consumption in patients undergoing uterine cancer surgery.

Furthermore, one study (49) has shown that dexmedetomidine can alleviate immune suppression and improve postoperative immune function by improving postoperative sleep and reducing postoperative sleep disturbances. An animal experiment shows that dexmedetomidine (50) effectively inhibits insulin-like growth factor 2 (IGF2) signaling pathway activation in ovarian cancer rats, thereby enhancing their immune function and suppressing the invasion and migration of ovarian cancer cells. Additionally, it has been shown that dexmedetomidine exerts systemic anti-inflammatory effects and alleviates immune suppression in patients undergoing modified radical mastectomy, with this effect being dose-dependent; medium to high doses (0.5 or 0.75 µg/kg) show more significant effects.

### 5.5 Nonsteroidal anti-inflammatory drugs

NSAIDs are among the most commonly used medications during the perioperative period. They inhibit cyclooxygenase (COX), reduce the synthesis of prostaglandins, thereby exerting anti-inflammatory and immunomodulatory effects. NSAIDs can also affect the activity of immune cells and regulate the production and release of cytokines involved in inflammation (51, 52). The administration of flurbiprofen during thoracoscopic radical surgery for lung cancer (53) can attenuate the levels of programmed cell death protein 1 (PD-1), an important immune checkpoint molecule, on CD8^+^ T cells within 72 hours postoperatively. PD-1 interacts with PD-L1 to promote immune escape in cancer (54). The reduction in PD-1 levels suggests that flurbiprofen may inhibit tumor immune escape. A longer-term follow-up study (55) indicated that postoperative analgesia with parecoxib sodium improves postoperative pain and immune function in patients undergoing hepatectomy for liver cancer, and may help delay tumor recurrence after surgery. Overall, the use of NSAIDs during the perioperative period provides benefits in pain management and controlling inflammatory responses. However, high doses or prolonged use may also exert immunosuppressive effects.

### 5.6 Local anesthetic

A study (56) conducted on animals found that intravenous injection of lidocaine can reduce postoperative lung metastasis in mice with breast cancer, possibly through its anti-inflammatory and anti-angiogenic effects. Clinically relevant concentrations of bupivacaine reduced the cell viability of ovarian (SKOV-3) and prostate (PC-3) cancer cells and inhibited cell proliferation and migration. It activated both intrinsic and extrinsic apoptotic pathways in ovarian cancer cells, while in prostate cancer cells, it only activated the intrinsic pathway, thereby demonstrating direct “anticancer” properties (57). Several studies (58–60) have demonstrated that intravenous infusion of lidocaine is beneficial both as a chemotherapy treatment and as an adjunct to chemotherapy in both perioperative and non-operative setting.

### 5.7 Other drugs

Dexamethasone, a glucocorticoid, is widely utilized during the perioperative period for preventing and treating allergic or hypersensitivity reactions, as well as for mitigating postoperative nausea and vomiting. An *in vitro* cell experiment (61) has shown found that dexamethasone upregulates the expression of CTLA-4 mRNA and protein in CD4 and CD8 T cells, blocking CD28-mediated cell cycle entry and differentiation, thereby inhibiting immune function.

Overall, commonly used anesthetic drugs have varying effects on the postoperative immune function of cancer patients. Therefore, these factors should be taken into consideration when optimizing anesthetic regimens. For more details, please refer to **Table 1**.

**Table 1:** Effects of anesthetics drugs on postoperative immune function in cancer patients and potential mechanisms.

Drugs	Effects on Immune Function	Mechanism
Opioid drugs (23–32)	Inhibit	Suppress the function of T and NK cells, regulation of cytokines and activation of the hypothalamic-pituitary-adrenal axis
Propofol (33–35)	Benefit	Related to its voltage-gated channels and anti-inflammatory effects
Midazolam (35, 36)	Have no significant impact	Both proinflammatory and anti-inflammatory
Etomidate (37, 38)	Have no significant impact	Inhibition of adrenal cortex function and anti-inflammatory effects
Ketamine (39, 40)	Inhibit	Decrease CD4 percentage, CD4/CD8 ratio and the levels of inflammatory cytokines
Inhalation Anesthetics (41–45)	Inhibit	Reduce the cytotoxicity of NK cells and increase pro-inflammatory factors, affect the tumor microenvironment and promote tumor cell invasion
Dexmedetomidine (46–50)	Benefit	Reduce the usage of anesthetic drugs especially opioids, implement anti-inflammatory and anti-stress measures, and improve postoperative sleep.
NSAIDs (51–55)	Benefit	Anti-inflammatory and immunomodulatory effects
Local anesthetic (56–60)	Benefit	Anti-inflammatory and anti-angiogenic effects, inhibited cancer cells proliferation and migration

## 6 Postoperative analgesia

Postoperative acute pain can impair immune function. Effective pain management can alleviate postoperative pain and stress, thereby enhancing postoperative immune function in cancer patients. Among different postoperative analgesia methods, epidural analgesia and nerve block analgesia can better maintain the immune function of patients (62). Intravenous analgesia has a stronger immunosuppressive effect on the body. The main reason is that intravenous analgesia often includes a certain amount of opioid drugs, which can inhibit immune function, and systemic circulation exacerbates the immunosuppressive effect of the body. In contrast, intrathecal analgesia or nerve block anesthesia utilizes the local effects of local anesthetics, which minimally affect the whole body. It effectively interrupts pain conduction, provides more effective analgesic effects, thereby alleviating the body’s stress response and improving postoperative immune function.

In postoperative intravenous analgesia, opioid drugs are the most commonly used. Despite their significant analgesic effects, they also have a substantial immunosuppressive effect. NSAIDs reduce prostaglandin synthesis to exert anti-inflammatory and immunomodulatory effects, while dexmedetomidine effectively alleviates inflammatory stress responses. Therefore, postoperative intravenous analgesia should avoid using opioid drugs alone as much as possible. Adopting multimodal analgesia strategies with reduced opioid drugs or the exclusion of opioid drugs, such as combining opioids with NSAIDs or dexmedetomidine, or combining NSAIDs with dexmedetomidine, can effectively improve postoperative immune function (63, 64).

## 7 Intraoperative blood transfusion

Cancer patients often present with varying degrees of anemia preoperatively, which is exacerbated by intraoperative blood loss during tumor resection, necessitating blood transfusion. However, perioperative blood transfusion is a double-edged sword. Although it effectively improves anemia or hypoalbuminemia and corrects coagulation disorders, it also suppresses postoperative immune function. A study (65) comparing the effects of allogeneic and autologous blood transfusion on immune function and prognosis in hepatocellular carcinoma patients found that autologous blood transfusion had a smaller impact on immune function compared to allogeneic transfusion. It even improved postoperative immune function and recurrence-free survival in these patients. Several studies (66, 67) have shown that intraoperative blood transfusion increases the incidence of postoperative infections and tumor recurrence in cancer patients, while reducing long-term survival rates. The mechanism by which blood transfusion causes postoperative immune suppression is not fully understood, but it is generally believed to involve exogenous tissue-compatible antigens, which increase prostaglandin E2 (PGE2) synthesis, inhibit monocytes, NK cells, and IL-2, alter lymphocyte subset ratios, and lead to the formation of specific antibodies and alloantibodies.

## 8 Body temperature

Perioperative temperature management plays a crucial role in the postoperative immune function of cancer patients. Hypothermia can suppress the immune system, increasing the risk of postoperative immune dysfunction and infectious complications. Shao et al. (68) divided patients undergoing open gastrectomy for gastric cancer into four groups: a normal temperature group and three groups with varying degrees of hypothermia. They monitored levels of TGF-β, IL-10, CD3^+^, CD4^+^/CD25^+^, and Treg cell counts. They found that lower body temperature was associated with greater suppression of postoperative immune function, and maintaining normal body temperature minimized changes in immune function. Temperature can have different effects on adaptive immunity at various levels; higher temperature generally promotes activation, function, and communication of immune cells, while lower temperature inhibits these processes (69).

## 9 Discussion

### 9.1 Human studies

The references cited in this manuscript include clinical human studies, animal studies and cell studies, with a predominant focus on human studies, which more directly reflect the relationship between anesthesia factors and immune function and prognosis in cancer patients. Human studies are conducted in actual clinical settings, providing a more accurate reflection of patient realities, making them crucial in evaluating the impact of anesthesia on cancer prognosis.

Many studies have suggested a correlation between anesthesia methods and the prognosis of cancer patients. However, a significant number of these studies lack stratified statistical analyses based on pathological grade, preoperative vascular invasion, and lymph node metastasis. Consequently, there remains considerable debate regarding the impact of anesthesia on prognosis in cancer surgery patients. Further research is needed to substantiate these findings and to explore the specific effects of anesthesia methods on the prognosis of patients with different pathological characteristics.

### 9.2 Animal and cell studies

Animal model studies and cell studies typically allow for more stringent control of variables and quicker results, which are particularly important in the initial exploratory stages. Through animal and cell research, the impact of specific anesthetics and surgical methods on immune function can be studied in detail in a controlled environment. Additionally, animal studies provide the opportunity for invasive procedures, enabling a deeper understanding of mechanisms and pathways.

However, animal models and cell studies also have significant limitations. There is no perfect non-human cancer model, and these models may lack many key biological steps of tumor development and metastasis. Furthermore, there are significant differences between animal and human immune systems, which can limit the translational applicability of the findings. Cell studies, while valuable for understanding basic mechanisms at a cellular level, cannot replicate the complex interactions present in a whole organism. Animal models and cell studies may not fully reflect the complex interactions between the immune system and tumors observed in human cancer patients.

## 10 Conclusion

In conclusion, it is evident that both surgery and anesthesia impact the postoperative immune function of cancer patients. However, current research has primarily focused on the effects within the first week after surgery, with limited studies and insufficient exploration of the longer-term effects on immune function. Future studies should focus on the long-term changes in postoperative immune function to gain a more comprehensive understanding of the impact of surgery and anesthesia on cancer patients, thereby guiding clinical management. Furthermore, their impact on the long-term prognosis of cancer patients is still debated. Although numerous retrospective studies have demonstrated that certain factors related to anesthesia can improve the prognosis of cancer patients, the complexity of factors influencing cancer prognosis means that there is currently insufficient evidence to draw definitive conclusions. Surgical procedures with minimal trauma-induced stress have less suppression on postoperative immune function. Among different anesthesia techniques, local anesthesia has the least impact on postoperative immune function, followed by nerve block anesthesia and spinal anesthesia. General anesthesia has the strongest immunosuppressive effect on postoperative immunity in cancer patients, with the immunosuppressive effect of sevoflurane inhalation anesthesia being stronger than that of propofol intravenous anesthesia. Among anesthetic drugs, those implicated in suppressing postoperative immune function include opioid analgesics, high doses of etomidate, ketamine, midazolam, and sevoflurane. Conversely, drugs that may potentially benefit or exhibit minimal suppression of postoperative immune function include propofol, melatonin, dexmedetomidine, and NSAIDs such as flurbiprofen. Additionally, effective postoperative analgesia can improve postoperative immune function, while intraoperative blood transfusion, especially allogeneic blood transfusion, perioperative hypothermia, and delayed emergence from general anesthesia, may inhibit the postoperative immune function of cancer patients.

In recent years, targeted therapy and immune checkpoint blockade for cancer have emerged as research hotspots in the field of tumor immunotherapy, particularly targeting immune checkpoints such as CTLA-4 and PD-1/PD-L1 (70). These therapeutic approaches aim to inhibit the immune escape of tumor cells and thereby achieve anti-tumor effects. Consequently, optimizing the selection and management of anesthesia and surgery during the perioperative period is crucial to maximize the preservation of postoperative immune function in cancer patients, mitigate the immune escape of residual cancer cells, and reduce the risk of postoperative tumor metastasis or recurrence, which is highly beneficial for cancer surgery patients. It is anticipated that in the near future, research on anesthesia and surgical factors related to tumor prognosis will become more comprehensive and in-depth, particularly through clinical follow-up studies on prognosis and long-term survival rates in a large sample of multicenter postoperative cancer patients. These studies will directly confirm their clinical effects, providing more direct and concrete evidence for the implementation of “perioperative anti-cancer anesthesia and surgical strategies” in clinical practice.

## 11 Strengths and limitations of this study

- This review provides a comprehensive and innovative assessment of the relationship between anesthesia, surgical factors and postoperative immune function in cancer patients.
- This review particularly comprehensively elucidates the relationship between various aspects of anesthesia and postoperative immune function in cancer patients, whereas previous studies have largely been confined to specific aspects.
- Language restrictions to English may have led to exclusion of additional studies.
